# Non-pulmonary vein mediated atrial fibrillation: A novel sub-phenotype

**DOI:** 10.1371/journal.pone.0184354

**Published:** 2017-09-07

**Authors:** Maureen Farrell, Zachary Yoneda, Jay Montgomery, Diane Crawford, Lauren Lee Wray, Meng Xu, Matthew J. Kolek, Travis Richardson, Ricardo Lugo, Mohamed Metawee, Greg Michaud, Juan Carlos Estrada, Pablo Saavedra, Sharon Shen, Arvindh Kanagasundram, Christopher R. Ellis, George Crossley, Dan Roden, M. Benjamin Shoemaker

**Affiliations:** 1 Department of Medicine, Vanderbilt University Medical Center, Nashville, Tennessee, United States of America; 2 Department of Biostatistics, Vanderbilt University, Nashville, Tennessee, United States of America; 3 Department of Biomedical Informatics, Vanderbilt University, Nashville, Tennessee, United States of America; 4 Department of Pharmacology, Vanderbilt University, Nashville, Tennessee, United States of America; University of Miami School of Medicine, UNITED STATES

## Abstract

**Background:**

Atrial fibrillation (AF) is a mechanistically heterogeneous disorder, and the ability to identify sub-phenotypes (“endophenotypes”) of AF would assist in the delivery of personalized medicine. We used the clinical response to pulmonary vein isolation (PVI) to identify a sub-group of patients with non-PV mediated AF and sought to define the clinical associations.

**Methods:**

Subjects enrolled in the Vanderbilt AF Ablation Registry who underwent a repeat AF ablation due to arrhythmia recurrence were analyzed on the basis of PV reconnection. Subjects who had no PV reconnection were defined as “non-PV mediated AF”. A comparison group of subjects were identified who had AF that was treated with PVI-only and experienced no arrhythmia recurrence >12 months. They were considered a group enriched for “PV-mediated AF”. Univariate and multivariable binary logistic regression analysis was performed to investigate clinical associations between the PV and non-PV mediated AF groups.

**Results:**

Two hundred and twenty nine subjects underwent repeat AF ablation and thirty three (14%) had no PV reconnection. They were compared with 91 subjects identified as having PV-mediated AF. Subjects with non-PV mediated AF were older (64 years [IQR 60,71] vs. 60 [52,67], P = 0.01), more likely to have non-paroxysmal AF (82% [N = 27] vs. 35% [N = 32], P<0.001), and had a larger left atrium (LA) (4.2cm [3.6,4.8] vs. 4.0 [3.3,4.4], P = 0.04). In univariate analysis, age (per decade: OR 1.56 [95% CI: 1.04 to 2.33], P = 0.03), LA size (per cm: OR 1.8 [1.06 to 3.21], P = 0.03) and non-paroxysmal AF (OR 8.3 [3.10 to 22.19], P<0.001) were all significantly associated with non-PV mediated AF. However, in multivariable analysis only non-paroxysmal AF was independently associated with non-PV mediated AF (OR 7.47 [95% CI 2.62 to 21.29], P<0.001), when adjusted for age (per decade: OR 1.25 [0.81 to 1.94], P = 0.31), male gender (OR 0.48 [0.18 to 1.28], P = 0.14), and LA size (per 1cm: 1.24 [0.65 to 2.33], P = 0.52).

**Conclusions:**

Non-paroxysmal AF was the only clinical variable found to be independently associated with non-PV mediated AF. We demonstrated that analysis of AF ablation outcomes data can serve as a tool to successfully identify a sub-phenotype of subjects who have non-PV mediated AF.

**Clinical trial registration:**

ClinicalTrials.gov ID # NCT02404415.

## Introduction

Atrial fibrillation (AF) is a common disease that affects over 6 million people in the United States [1]. Like other multifactorial inheritance disorders, AF is thought to develop when a sufficient burden of acquired factors (comorbidities) are present in a genetically susceptible individual. Great strides have been made toward discovering the pathophysiology of AF, and it is now understood that numerous molecular pathways involved in a variety of functions such as atrial fibrosis, ion channel function, autonomic innervation, myocyte coupling, and others converge to promote AF triggers and/or a pro-arrhythmic substrate in the left atrium (LA), right atrium (RA), and pulmonary veins (PVs) [2].

Over the past decade, pulmonary vein isolation (PVI) has been expanded from its original use in relatively young healthy patients with early-onset paroxysmal AF to include older patients with cardiopulmonary diseases and non-paroxysmal (persistent and long-standing persistent) AF [1]. This experience has confirmed that isolating the PV myocardial sleeves can effectively treat AF in many patients with a wide range of clinical profiles but also presents a challenge in predicting which patients have AF due primarily to PV triggers. Recently, it has been recognized that up to 41% of subjects with recurrent AF who were treated with AF ablation have no PV reconnection observed at the time of a repeat ablation procedure [3]. These patients can be considered to have non-PV mediated AF and represent an interesting AF subgroup. We believe that an opportunity exists to learn more about AF pathogenesis by recognizing a novel AF sub-phenotype (“endophenotype”) defined by clinical response to PVI:”Non-PV mediated AF”. Accordingly, the main goals of this study were to: 1) define the clinical characteristics of subjects with non-PV mediated AF by examining a large cohort of subjects with recurrent AF undergoing repeat ablation and analyzing them based on the presence of PV reconnection; 2) identify a comparison group of patients enriched for PV-mediated AF who underwent PVI-only and had long-term freedom from recurrence of AF; and 3) perform a case/control analysis to define the clinical associations with non-PV mediated AF.

## Methods

### Study cohort

Subjects were enrolled in the Vanderbilt Atrial Fibrillation Ablation Registry (VAFAR) and underwent catheter-based ablation for non-valvular AF between 2003 and 2015. VAFAR is a prospective, observational clinical biorepository [4]. Subject characteristics, ablation details, and follow-up data were recorded in a central database (REDCap) [5]. All subjects provided written informed consent for the study, and the study was approved by the Vanderbilt University Institutional Review Board.

Age at ablation was defined as the subject’s age at the time of the *de-novo* ablation procedure. Subjects were defined as having non-paroxysmal AF if they ever had an episode of AF lasting ≥ 7 days or an AF episode that necessitated termination with direct current or pharmacologic cardioversion. Subjects were identified as having lone AF if they were <60 years of age and had no cardiopulmonary comorbidities or structural heart disease at the time of ablation. Left atrial (LA) size was recorded as the diameter measured in the anteroposterior dimension. Left ventricular ejection fraction was analyzed as a continuous variable with the upper limit truncated at 55%. Comorbid conditions (hypertension, diabetes mellitus, congestive heart failure, coronary artery disease) were recorded as present if the subject carried the clinical diagnosis at the time of ablation. Pulmonary vein anatomy was assessed by pre-ablation imaging with a cardiac MRI or CT angiogram. Antiarrhythmic drug use was defined according to the Vaughan-Williams Classification system with the exception of amiodarone, which was reported as a separate category due to its overlapping mechanisms of action with multiple antiarrhythmic drug classes. Additional LA ablation was defined as any ablation performed in the left atrium other than pulmonary vein isolation (i.e. mitral isthmus line, roof line, inferior line, complex fractionated atrial electrogram ablation, focal atrial tachycardia ablation, targeting of low voltage areas, etc). An arrhythmia recurrence was defined according to standards established by the Heart Rhythm Society, European Heart Rhythm Association, and the European Cardiac Arrhythmia Society as any episode of AF, atrial tachycardia, or atrial flutter lasting greater than 30 seconds and occurring after a 3-month blanking period [6].

AF ablation was performed using contemporary tools and either radiofrequency or cryoballoon techniques. AF ablation was performed under general anesthesia with continuous invasive monitoring of blood pressure and oxygen saturation. Transseptal access was obtained using intracardiac or transesophageal echocardiographic guidance. Anticoagulation with heparin was used to maintain an activated clotting time greater than 300 seconds during left atrial (LA) access. For radiofrequency ablation, a 3-dimensional mapping system (Carto, Biosense-Webser, Inc., Diamond Bar, CA; NavX-Ensite, Endocardial Solutions, Inc., St. Paul, MN) was used for non-fluoroscopic catheter navigation, computed tomographic or magnetic resonance image integration, and tagging of ablation sites. An irrigated-tip ablation catheter was used. Circumferential LA ablation lines were placed around the antrum of the ipsilateral pulmonary veins with power of 25–30 watts along the posterior wall and 30–40 watts along the floor, anterior wall, and roof. Empiric linear lesions were added to the LA roof, basal posterior wall, and mitral isthmus in selected patients based on operator discretion. Complex fractionated electrogram mapping was not routinely performed. For ablations using the cryoballoon (Arctic Front, Medtronic Inc., Minneapolis, MN), a complete seal was confirmed using contrast injection, and in some cases pressure measurement, followed by circumferential PV antral isolation using two 3 minute freeze cycles. After circumferential line placement, bidirectional block was tested using a multipolar circular mapping catheter with additional ablation performed as needed to achieve complete block.

#### Identification of non-PV mediated AF cases

To identify cases of non-PV mediated AF and investigate clinical associations with PV reconnection, subjects were selected who: 1) experienced an arrhythmia recurrence after a *de-novo* AF ablation procedure; and 2) underwent a repeat AF ablation procedure. At the time of the repeat AF ablation procedure, a multipolar mapping catheter was placed in each PV. The presence of conducted PV potentials was used to indicate electrical PV reconnection and care was taken to search the proximal aspect of each PV to prevent missing evidence of PV reconnection due to placing the catheter too distally into the PV. The presence or absence of reconnection was recorded for each PV. Differential pacing maneuvers were used to distinguish far field left atrial appendage and superior vena cava signals from PV potentials in the left upper PV (LUPV) and right upper PV (RUPV), respectively. Subjects who had no PV reconnection observed at that time of repeat ablation were defined as having non-PV mediated AF.

#### Identification of a comparison group enriched for PV-mediated AF

As a comparison group for non-PV mediated AF, a group of subjects enriched for PV-mediated AF was selected from VAFAR based on the following eligibility criteria: 1) at the time of *de-novo* catheter-based AF ablation, PVI was performed without any other adjuvant ablation in the LA or RA (i.e. no additional LA linear or focal ablation, no CTI ablation, etc); 2) post-ablation follow-up was for ≥12 months and no arrhythmia recurrence was observed. Post-ablation monitoring for arrhythmia recurrence included a 12-lead ECG and use of an ambulatory ECG monitor at 3, 6, and 12-months. Additional monitoring was used as necessary for patient report of symptoms suggestive of recurrence.

## Statistical analysis

Continuous variables were expressed as the median and interquartile range (IQR) and categorical variables were presented as the frequency and percentage. Descriptive statistics for baseline characteristics were presented for the overall cohort, and also stratified between subjects who had no PVs reconnected compared with subjects who had ≥1 PV reconnected. Groups were compared using a Chi-square test for categorical variables and a Mann-Whitney U test for continuous variables. Univariate and multivariable ordinal logistic regression (ordered logit) models were used to test for associations between clinical variables and the number of PVs reconnected. A ratio of 15:1 was selected for number of subjects per degree of freedom in order to avoid overfitting the multivariable ordinal logistic regression model. All covariates included in the multivariable model were pre-defined. Age and gender were included as standard covariates for multivariable analysis and non-paroxysmal AF and lone AF were selected based on an *a-priori* expected relationship to reconnection. To investigate whether the presence of covariates associated with non-PV mediated AF confounded the association with the number of PVs reconnected, a sensitivity analysis was performed removing subjects with no PVs reconnected so that an ordinal regression model of number of PVs equal to 1–4 was performed. Next, a multivariable binary logistic regression model was used to test for an association between subjects with PV reconnection (yes or no). All covariates were pre-defined in this model. Age and gender were included as standard covariates for multivariable analysis and non-paroxysmal AF and LA size were selected based on an *a-priori* expected relationship with non-PV mediated AF triggers/substrate. A ratio of 8:1 was selected for number of subjects to degrees of freedom to avoid over-fitting the binary logistic regression model. Finally, we compared subjects who had no PV reconnection at the time of repeat AF ablation (non-PV mediated AF) to the comparator PV-mediated AF group. Baseline characteristics were compared using a Mann-Whitney U test or Chi-square test. Univariate and multivariable binary logistic regression models were used to test for an association between clinical variables and non-PV mediated versus PV-mediated AF. To examine whether the association remained after exclusion of subjects who had only an AT/AFL recurrence, a sub-group analysis was performed excluding those subjects. A P-value <0.05 was considered statistically significant for all comparisons. Analyses and figures were generated using a variety of statistical packages including R package version 3.3.0 (https://www.r-project.org/), SPSS version 24 (IBM Corp., Armonk, NY), and GraphPad Prism version 5.04 (GraphPad Software, Inc., La Jolla, CA).

## Results

### Analysis of the repeat ablation cohort

**Fig 1:**
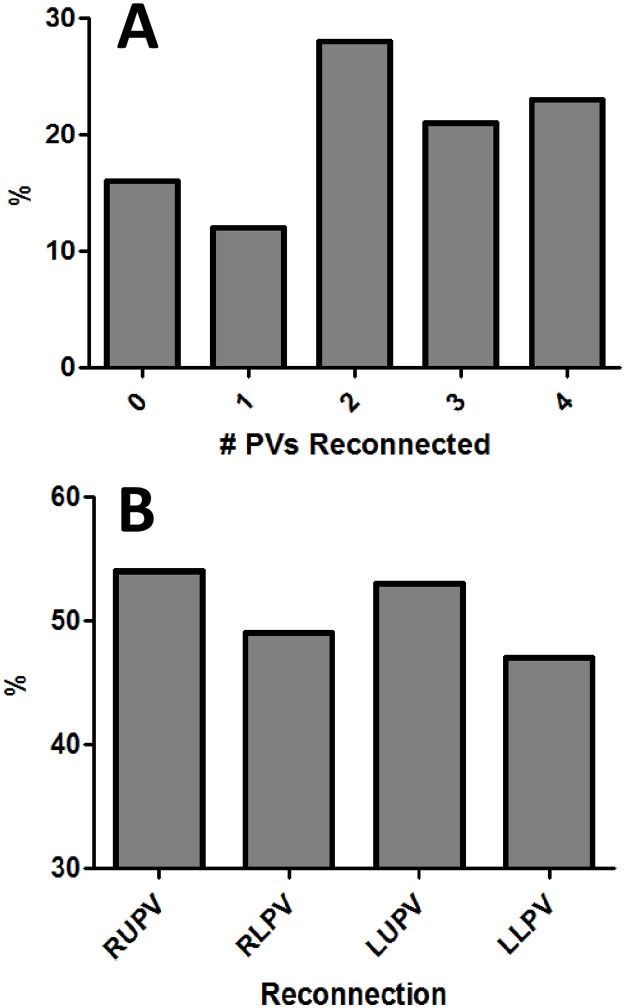
**Panel A** displays the proportion of subjects according to the number of PVs found to be reconnected at the time of repeat AF ablation. **Panel B** displays the proportion of PV reconnection per specific PV (RUPV = right upper PV, RLPV = right lower PV, LUPV = left upper PV, LLPV = left lower PV).

Nine hundred and eighty seven patients underwent a *de-novo* AF ablation, and 439 (44%) had a documented arrhythmia recurrence by 12-months). From the *de-novo* ablation cohort, 539 (55%) had non-paroxysmal AF, and 261 (48%) of the non-paroxysmal AF subjects experienced a qualifying arrhythmia recurrence. From the subjects that recurred, 229 underwent a first repeat AF ablation procedure and met eligibility criteria. The median age was 61 years (IQR 55,67) and 65% of subjects were male (Table 1). Thirty three subjects (33/229; 14%) were found to have no PV reconnection at the time of repeat AF ablation and were identified as cases of non-PV mediated AF. Compared to subjects with ≥1 PV reconnected, subjects without PV reconnection were older (64 years [60,71] vs. 61 [55,67], P = 0.009) and more likely to have non-paroxysmal AF (82% [N = 27] vs. 50% [N = 98], P<0.001). Within subjects who underwent de-novo AF ablation with PVI-only (N = 322), 43% (N = 137) had non-paroxysmal AF, and 53% (N = 73) of those subjects experienced AF recurrence. The median number of PVs reconnected per subject was 2 (1,3), and a breakdown of the frequency of reconnection per specific PV is presented in Fig 1.

**Table 1:** Characteristics of subjects with AF recurrence undergoing repeat ablation.

	Overall (N = 229)	No PV Reconnection (N = 33)	PV Reconnection (N = 196)	P-value
Age at Ablation (years)	61 (55,67)	64 (60,71)	61 (55,67)	0.009*
Male gender (%)	148 (65%)	21 (64%)	127 (65%)	0.90
Race White Black Other	- 219 (96%) 5 (2%) 5 (2%)	- 32 (97%) 1 (3%) 0 (0%)	- 187 (95%) 4 (2%) 5 (3%)	0.43
Ethnicity Non-Hispanic Hispanic	- 229 (100%) 0 (0%)	- 33 (100%) 0 (0%)	- 196 (100%) 0 (0%)	—
Height (cm)	178 (168,183)	178 (170,185)	175 (167,183)	0.498
Body Mass Index (kg/m^2^)	30 (27,36)	31 (26,34)	30 (27,36)	0.384
Non-Paroxysmal AF	125 (55%)	27 (82%)	98 (50%)	<0.001*
Lone AF	35 (15%)	4 (12%)	31 (16%)	0.58
Time Since AF Diagnosis (years)	3.8 (1.3,8.1)	4.8 (1.0,8.0)	3.8 (1.4,8.1)	0.714
Hypertension	158 (69%)	27 (82%)	131 (67%)	0.73
Diabetes	53 (23%)	10 (30%)	43 (22%)	0.30
Congestive Heart Failure	23 (10%)	4 (12%)	19 (10%)	0.68
Coronary Artery Disease	82 (36%)	10 (30%)	72 (37%)	0.47
Left Atrial Size (cm)	4.1 (3.6,4.7)	4.2 (3.6,4.8)	4.0 (3.6,4.7)	0.459
Left Ventricular Ejection Fraction (%)	55 (55,55)	55 (54,55)	55 (55,55)	0.635
Pulmonary Vein Anatomy 4 separate PVs Common Left PV Common Right PV Common Left and Right PV	- 214 (94%) 12 (5%) 1 (<1%) 0 (0%)	- 29 (91%) 3 (9%) 0 (0%) 0 (0%)	- 185 (94%) 9 (5%) 1 (<1%) 0 (0%)	0.51
Accessory Pulmonary Vein (yes/no)	36 (16%)	5 (15%)	31 (16%)	0.91
Common Pulmonary Vein (yes/no)	13 (6%)	3 (9%)	10 (5%)	0.35
Antiarrhythmic Drug Use Prior to Ablation None Class I Class III Amiodarone	- 32 (14%) 49 (21%) 95 (42%) 44 (19%)	- 5 (15%) 7 (21%) 12 (36%) 9 (27%)	- 27 (14%) 42 (23%) 83 (44%) 35 (19%)	0.70
Pulmonary Vein Isolation Technique Radiofrequency Cyroballoon	- 205 (90%) 24 (10%)	- 29 (88%) 4 (12%)	- 176 (90%) 20 (10%)	0.74
Additional Left Atrial Ablation Ϯ	67 (29%)	8 (24%)	59 (30%)	0.49
Cavotricuspid Isthmus Ablation	72 (31%)	14 (42%)	58 (30%)	0.15
Type of Arrhythmia Recurrence AF only AF and AFL or AT AFL or AT only	- 158 (69%) 23 (10%) 36 (16%)	- 23 (72%) 2 (6%) 7 (21%)	- 135 (73%) 21 (11%) 29 (16%)	0.51
Time to Arrhythmia Recurrence (days)	86 (26,255)	145 (68,283)	85 (24,252)	0.157
Time from First Ablation to Repeat (days)	476 (249,980)	469 (254,953)	481 (248,987)	0.886

^Ϯ^mitral lines, roof lines, other LA linear ablation, complex fractionated atrial electrogram ablation.

* p = < .05

**Fig 2:**
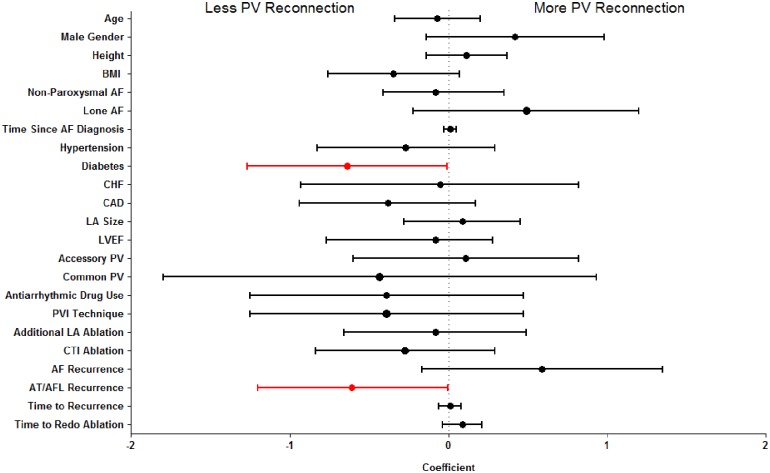
Presents the association with PV reconnection analyzed by univariate ordinal regression for subjects with 1–4 PVs reconnected. Negative coefficients indicate an association with less PV reconnection and positive coefficients indicate an association with more PV reconnection. P<0.05 is displayed in red.

Univariate ordinal regression analysis exploring the association between clinical variables and the number of PVs reconnected demonstrated non-paroxysmal AF (β = -0.66 [95% CI -1.15 to -0.17], P = 0.008) and diabetes (β = -0.57 [-1.14 to -0.01], P = 0.045) were significantly associated with fewer reconnected PVs (S1 Table). Furthermore, non-significant trends were observed for an association between older age (per decade: β = -0.23 [-0.48 to -0.01), P = 0.06) and hypertension (β = -0.49 [-1.01 to -0.03], P = 0.06) with fewer reconnected PVs. In multivariable adjustment, only non-paroxysmal AF remained significantly associated with the number of PVs reconnected (β = -0.79 [-1.35 to -0.23], P = 0.006). Due to concern that including subjects with no PVs reconnected would prevent associations with PV reconnection being detected due to confounding from factors associated with non-PV mediated AF, a sensitivity analysis was performed that restricted the number of PVs reconnected to between 1–4. This analysis found that non-paroxysmal AF was no longer associated with PV reconnection (univariate analysis: P = 0.51, Multivariable analysis: P = 0.64). The sensitivity analysis did detect a significant univariate association between fewer PVs reconnected and diabetes (β = -0.64 [95% CI: -1.27 to -0.01], P = 0.047) and subjects who experienced an AT/AFL recurrence following their first ablation (β = -0.62 [-1.2 to -0.003], P = 0.049) (Fig 2). No significant associations were detected in our pre-specified multivariable model (S2 Table), but a post-hoc analysis demonstrated the association between AFL/AT recurrence and fewer PVs reconnected remained significant with multivariable adjustment (β = -0.82 [-1.48 to -0.17], P = 0.014).

Univariate binary logistic regression analysis examining the association between clinical variables and PV reconnection (yes/no) demonstrated that age (per decade: OR 0.6 [95% CI 0.39 to 0.92], P = 0.02) and non-paroxysmal AF (OR 0.22 [0.09 to 0.56], P<0.001) were significantly associated with reduced odds of PV reconnection, however, in multivariable analysis only non-paroxysmal AF remained significant (OR 0.25 [0.10 to 0.64], P = 0.004) (Table 2). The sub-group analysis excluding subjects who experienced only an AT/AFL recurrence demonstrated the association between non-paroxysmal AF and non-PV mediated AF remained highly significant (OR 6.15 [2.24 to 16.85], P<0.001).

**Table 2:** Association between Baseline characteristics and PV reconnection (yes/no).

	Univariate Analysis		Multivariable Analysis	
	OR (95% CI)	P-value	OR (95% CI)	P-Value
Age (per decade)	0.60 (0.39–0.92)	0.02*	0.64 (0.39–1.04)	0.07
Male gender	1.05 (0.49–2.23)	0.90	0.93 (0.41–2.12)	0.86
Non-Paroxysmal AF	0.22 (0.09–0.56)	<0.001*	0.25 (0.10–0.64)	0.004*
Lone AF	1.36 (0.45–4.15)	0.59	0.81 (0.24–2.74)	0.74

* p = < .05

### Analysis of PV-mediated AF versus non-PV mediated AF

**Fig 3:**
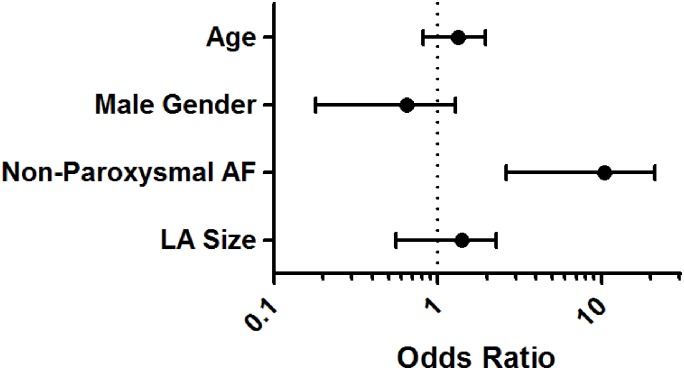
Non-paroxysmal AF (OR 7.47 [95% CI 2.62 to 21.29], P<0.001) is independently associated with non-PV mediated AF when adjusted for age (per decade), gender, and LA size (per cm).

Ninety one subjects were identified who met eligibility for inclusion in the group enriched for PV-mediated AF to be compared with the 33 subjects in the non-PV mediated AF group. Subjects with non-PV mediated AF were older (64 years [IQR 60,71] vs. 60 [52,67], P = 0.01), more likely to have non-paroxysmal AF (82% [N = 27] vs. 35% [N = 32], P<0.001), and had a larger LA (4.2cm [3.6,4.8] vs. 4.0 [3.3,4.4], P = 0.04) than subjects in the comparator PV-mediated AF group (Table 3). In univariate regression analysis, older age, non-paroxysmal AF, and larger LA size were all significantly associated with increased odds of having non-PV mediated AF. In multivariable analysis, non-paroxysmal AF was associated with greater than a 7-fold increased odds of having non-PV mediated AF (OR 7.47 [95% CI 2.62 to 21.29], P<0.001), and was the only covariate that remained significant when adjusted for age (OR 1.25 [0.81 to 1.94], P = 0.31), male gender (OR 0.48 [0.18 to 1.28], P = 0.14), and LA size (per 1cm: 1.24 [0.65 to 2.33], P = 0.52) (Fig 3, S3 Table).

**Table 3:** Comparison of pulmonary vein and non-pulmonary vein mediated AF.

	Non-PV mediated AF (N = 33)	PV-mediated AF (N = 91)	P-value
Age at First Ablation (years)	64 (60,71)	60 (52,67)	0.01*
Male gender (%)	21 (64%)	69 (76%)	0.19
Race White Black Other	- 32 (97%) 1 (3%) 0 (0%)	- 89 (98%) 2 (2%) 0 (0%)	0.79
Ethnicity Non-Hispanic Hispanic	- 33 (100%) 0 (0%)	- 90 (99%) 1 (1%)	0.43
Height (cm)	178 (170,185)	179 (170,185)	0.73
Body Mass Index (kg/m^2^)	31 (26,34)	29 (26,33)	0.72
Non-Paroxysmal AF	27 (82%)	32 (35%)	<0.001*
Lone AF	4 (12%)	16 (18%)	0.45
Time Since AF Diagnosis (years)	4.8 (1.0,8.0)	3.4 (1.3,6.2)	0.64
Hypertension	27 (82%)	60 (66%)	0.08
Diabetes	10 (30%)	14 (15%)	0.07
Congestive Heart Failure	4 (12%)	4 (4%)	0.14
Coronary Artery Disease	10 (30%)	32 (35%)	0.61
Left Atrial Size (cm)	4.2 (3.6,4.8)	4.0 (3.3,4.4)	0.04*
Left Ventricular Ejection Fraction (%)	55 (54,55)	55 (55,55)	0.21
Pulmonary Vein Anatomy 4 separate PVs Common Left PV Common Right PV Common Left and Right PV	- 29 (91%) 3 (9%) 0 (0%) 0 (0%)	- 84 (92%) 4 (4%) 1 (1%) 0 (0%)	0.66
Accessory Pulmonary Vein (yes/no)	5 (15%)	15 (17%)	0.86
Common Pulmonary Vein (yes/no)	3 (9%)	5 (6%)	0.45
Antiarrhythmic Drug Use Prior to Ablation None Class I Class III Amiodarone	- 5 (15%) 7 (21%) 12 (36%) 9 (27%)	- 10 (11%) 31 (35%) 33 (37%) 15 (17%)	0.38
Pulmonary Vein Isolation Technique Radiofrequency Cyroballoon	- 29 (88%) 4 (12%)	- 82 (90%) 9 (10%)	0.72

* p = < .05

## Discussion

In the era of precision medicine, mechanistically heterogeneous disorders such as AF will need to be sub-phenotyped for the successful deployment of targeted therapies. Given the effectiveness of PVI for treating some patients with AF, we demonstrated how the clinical response to PVI may provide a tool to easily identify a subgroup of patients who have non-PV mediated AF and respond poorly to PVI alone. In a relatively large cohort of repeat ablation patients, we found that 14% have AF despite no reconnection of their PVs. This scenario strongly suggests the existence of AF triggers and/or a pro-arrhythmic atrial substrate localizing to the body of the LA or the RA as the predominant AF mechanism. The presence of PV reconnection in a patient with arrhythmia recurrence does not definitively indicate that the source of arrhythmia recurrence localizes to the PV’s because a non-PV source of AF could co-exist in a patient with reconnection. Therefore, as a comparison group, we identified subjects who underwent an AF ablation procedure consisting of PVI without ablation of any other targets in the LA or RA and had long-term freedom from AF recurrence. These patients were believed to represent a group enriched for AF sources localizing to the PV myocardial sleeve and/or antrum as their predominant AF mechanism. Subjects with non-PV mediated AF were older, more likely to have non-paroxysmal AF, and had larger LA. While these are all well recognized predictors of recurrence following AF ablation [7–8], our results: 1) confirm the idea that a common etiology for recurrence is the existence of non-PV sources of AF rather than entirely a result of PV reconnection; and 2) demonstrate non-paroxysmal AF is the most powerful clinical predictor of non-PV mediated AF as it was the only covariate that remained statistically significant in multivariable analysis and conferred an effect size of > 7-fold.

### Comparison to the published literature

Recently, several high-volume ablation centers have published clinical series describing the prevalence and clinical management of patients with non-PV mediated AF. It is speculated that with advances in catheter technology and improvement in techniques for PVI, the detection of subjects with non-PV mediated AF will become increasingly common [3]. This trend is reflected in the prevalence rate of non-PV mediated AF observed in these reports. In Sadek et al., ablation records from 2003 to 2013 were examined and the rate of non-PV mediated AF was 5%, whereas more recent reports by Kim et al. and Baldinger et al. reported rates of non-PV mediated AF to be 36% and 41%, respectively [3, 9–10]. A systematic review by Nery et al., which included studies ranging from 2003 to 2016, reported an overall rate of 9% for non-PV mediated AF [11]. Taken together, the rate of 14% observed in our study is consistent with the other published reports over the time frame we included (2003–2015). There was general agreement among these authors that non-PV mediated AF was a unique population for which the pathophysiology was not related to PV-triggers, the rate of subsequent recurrence was higher, and the optimal ablation strategy of targeting non-PV sources (focal trigger ablation, empiric linear ablation, and/or substrate ablation) was unknown and should be an area of future investigation.

### Consideration of type of recurrence on results

The distinction between subjects who have recurrence of AF following AF ablation compared to those that recur with only AT or AFL is important to consider in the context of attributing the recurrence to pre-existing non-PV sources. It has been previously shown that the addition of empiric linear ablation may be pro-arrhythmic due to electrical gaps leading to reentry [12–14]. This suggests that AT/AFL recurrences are iatrogenic and should be excluded from an analysis of non-PV mediated AF. However, similar to electrical reconnection of the PVs, which has been found to be present in up to 59% of patients who remain AF-free following ablation [11], electrical gaps in linear ablation lines are likely present in the majority of patients and may not alone be sufficient to produce an AT/AFL recurrence without a trigger. Therefore, our primary analysis included subjects with both AF and AT/AFL recurrences. However, we investigated the possibility that AT/AFL-only recurrences should not be included by performing a sub-group analysis, which found the association between non-paroxysmal AF and non-PV mediated AF remained highly significant after removing the subjects who had only an AT/AFL recurrence.

## Limitations

A potential limitation is that the relationship observed between non-paroxysmal AF and the number of PVs reconnected is likely confounded by the strong association detected between non-paroxysmal AF and non-PV mediated AF. To address this possibility we performed a sensitivity analysis by removing subjects who had no PV reconnection, thereby restricting the analysis to only subjects with one to four PVs reconnected. Doing so resulted in non-paroxysmal AF no longer being significantly associated with PV reconnection, supporting our idea that non-paroxysmal AF is predominantly associated with non-PV mediated AF rather than mechanisms which may drive reconnection of the pulmonary veins. Another potential limitation is the lack of a standardized approach to the use of empiric linear ablation. This was done at the operators discretion, which represented a variety of evolving practice patterns given the subjects included in this analysis underwent ablation by 10 different operators over a 12-year period. It is unclear whether a bias could have existed in the use of linear ablation that affected our results. Also, the sample sizes used in this study were modest compared to the norms of the AF ablation literature in-general; however, a repeat ablation cohort was required to study non-PV mediated AF. Only 20–30% of AF ablation subjects undergo a repeat procedure [15], therefore 229 repeat AF ablation patients represents a relatively large sample. Finally, this study is limited because it is a single center study and differences between centers may exist that affect the generalizability of our findings such as the clinical characteristics of the study population, patient follow-up, and/or operator technique.

## Clinical implications and future directions

A shortcoming of outcomes research that uses AF recurrence as the primary endpoint is that it does not provide evidence to support a specific etiology for recurrence. We believe our approach presents an easy method to identify cases of non-PV mediated and, by performing a case/control analysis with subjects without recurrence who underwent PVI-only, may provide a novel approach to specifically study one of the major etiologies for AF recurrence post-ablation. As a first step, we found that non-paroxysmal AF was the only clinical predictor of non-PV mediated AF, and it was significant with a large effect size (OR >7, P = 0.006). In the future, with major advances continuing in the discovery of the genetic basis of AF [16], the identification of novel AF sub-phenotypes (i.e. non-PV mediated AF) may enable additional genetic association testing to complement efforts in the field of functional genomics aimed at providing clues as to the mechanism of newly identified genetic variants.

## Conclusions

Analysis of AF ablation outcomes data can serve as a tool to identify subjects who have non-PV mediated AF—a novel AF sub-phenotype. Using this technique, we demonstrated that non-paroxysmal AF was the only clinical variable independently associated with non-PV mediated AF. Future studies may be able to use this sub-phenotype to help localize the mechanism of AF when investigating newly discovered clinical and genetic associations.

## Supporting information

S1 Table Supplemental table 1. Results of Ordinal Regression Analysis on Number of PVs Reconnected (0–4).
(TIF)

S2 Table Supplemental table 2. Sensitivity Analysis: Ordinal Regression on Number of PVs Reconnected (1–4).
(TIF)

S3 Table Supplemental table 3. Logistic Regression Testing Associations with Non-PV mediated AF.
(TIF)
