# Supplementary figures and images for: Non-pulmonary vein mediated atrial fibrillation: A novel sub-phenotype
Source: PLoS One. 2017 Sep 7;12(9):e0184354. doi: 10.1371/journal.pone.0184354 (PMC5589236; doi:10.1371/journal.pone.0184354)

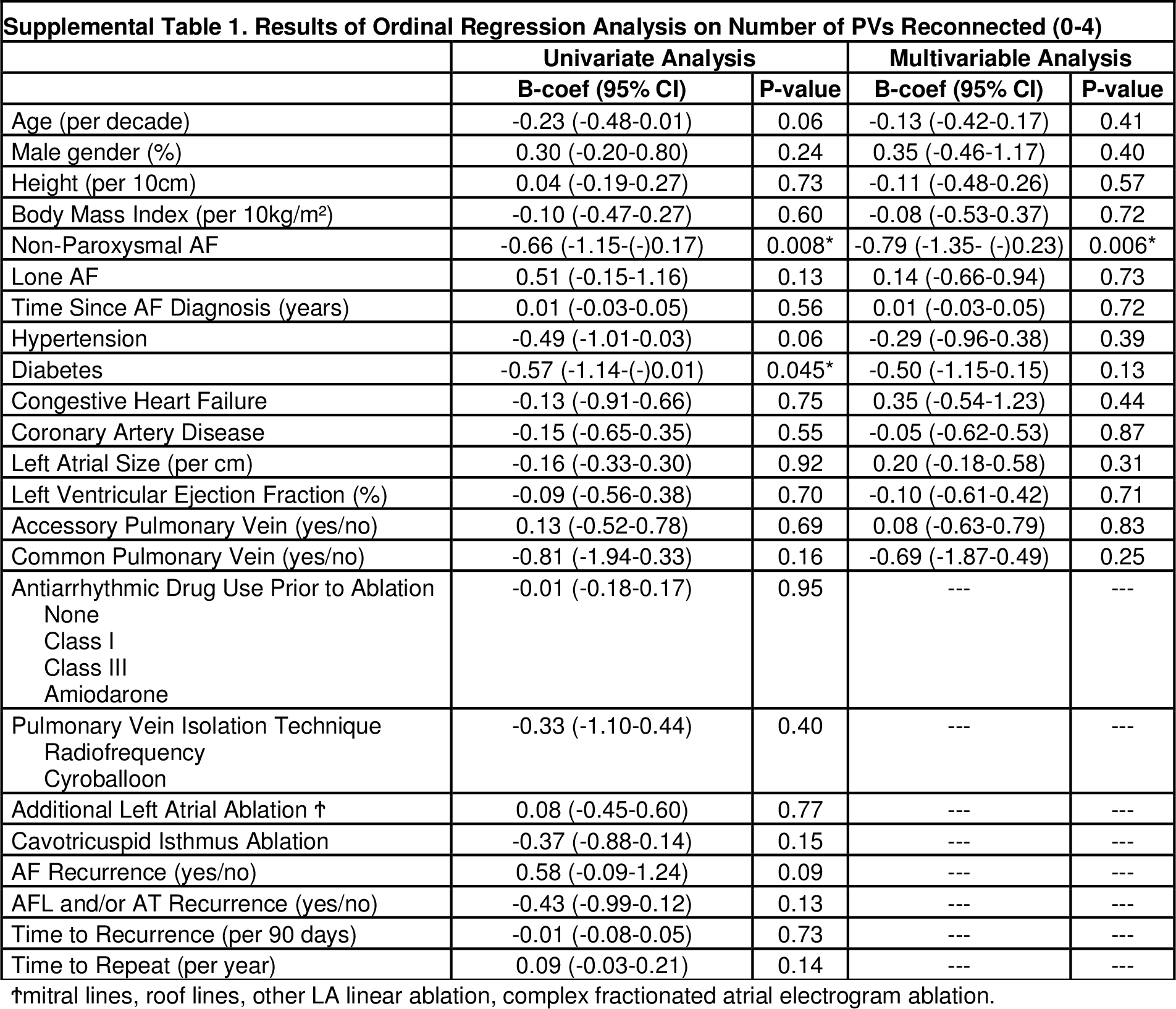

Supplement: S1 Table — Results of Ordinal Regression Analysis on Number of PVs Reconnected (0–4). (TIF) [file pone.0184354.s001.tif]

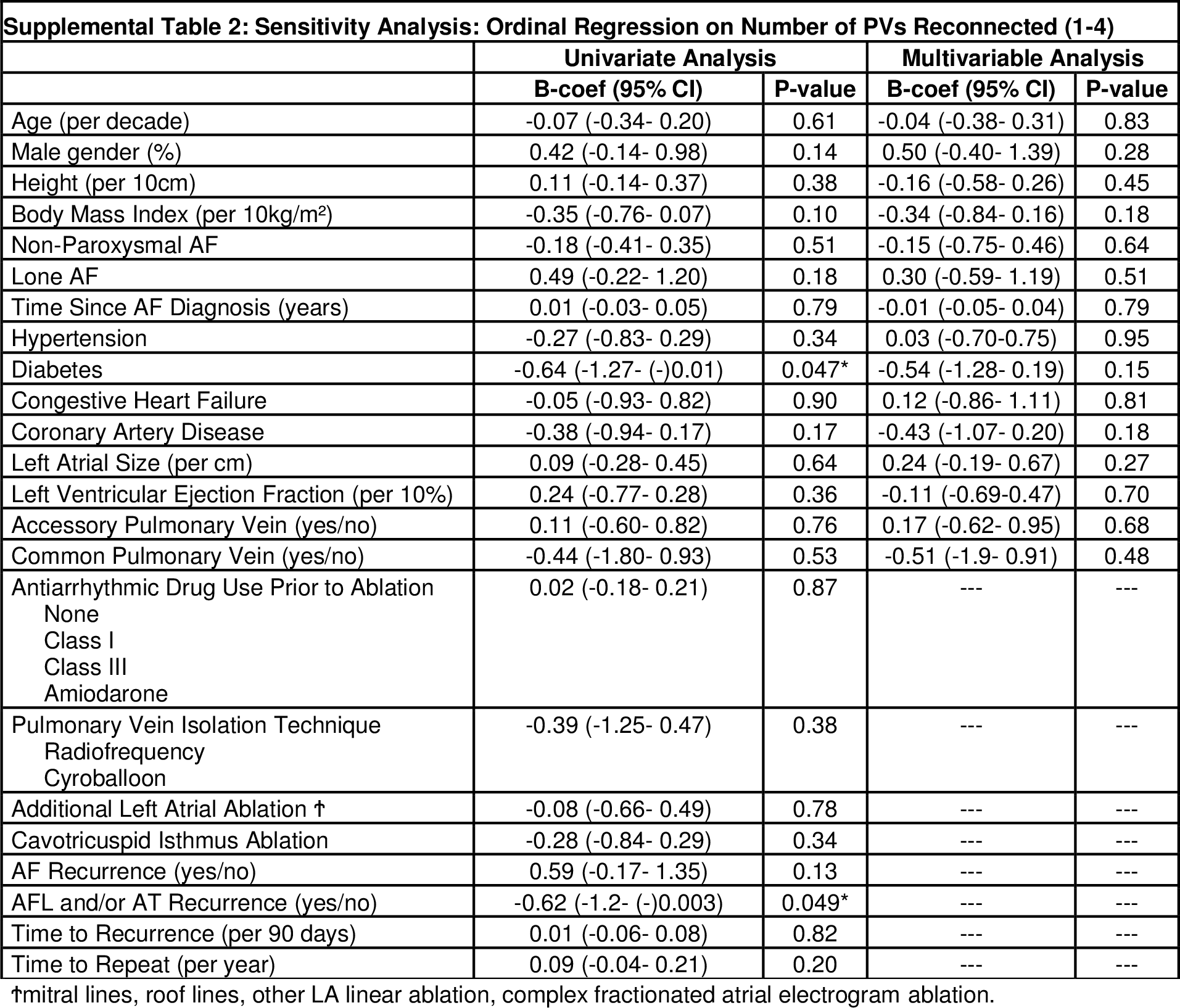

Supplement: S2 Table — Sensitivity Analysis: Ordinal Regression on Number of PVs Reconnected (1–4). (TIF) [file pone.0184354.s002.tif]

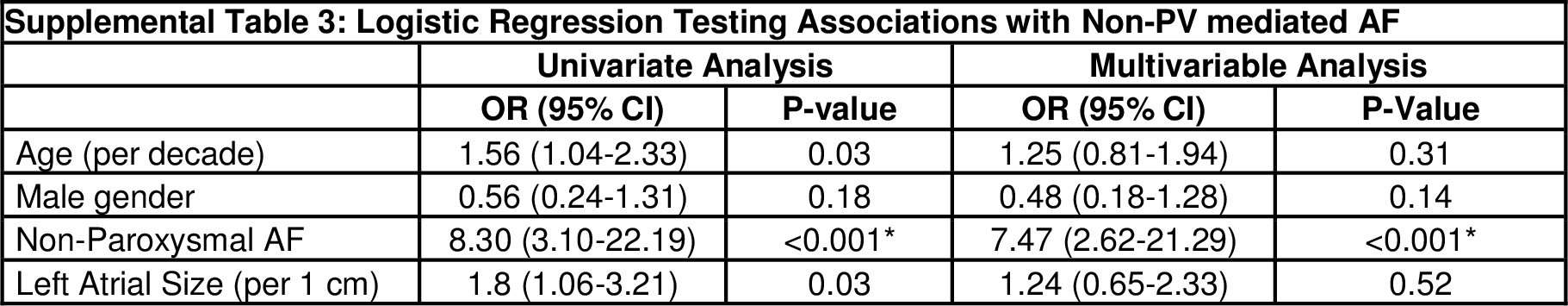

Supplement: S3 Table — Logistic Regression Testing Associations with Non-PV mediated AF. (TIF) [file pone.0184354.s003.tif]
